# Vector competence and transovarial transmission of two *Aedes aegypti* strains to Zika virus

**DOI:** 10.1038/emi.2017.8

**Published:** 2017-04-26

**Authors:** Chun-xiao Li, Xiao-xia Guo, Yong-qiang Deng, Dan Xing, Ai-juan Sun, Qin-mei Liu, Qun Wu, Yan-de Dong, Ying-mei Zhang, Heng-duan Zhang, Wu-chun Cao, Cheng-feng Qin, Tong-yan Zhao

**Affiliations:** 1 Department of Vector Biology and Control, State Key Laboratory of Pathogen and Biosecurity, Institute of Microbiology and Epidemiology, Beijing 100071, China; 2 Department of Virology, State Key Laboratory of Pathogen and Biosecurity, Institute of Microbiology and Epidemiology, Beijing 100071, China; 3 Department of Epidemiology, State Key Laboratory of Pathogen and Biosecurity, Institute of Microbiology and Epidemiology, Beijing 100071, China

**Keywords:** *Aedes aegypti*, transovarial transmission, vector competence, Zika virus

## Abstract

Zika virus (ZIKV) has become a serious threat to global health since the outbreak in Brazil in 2015. Additional Chinese cases have continuously been reported since the first case of laboratory-confirmed ZIKV infection in China on 6 February 2016. *Aedes aegypti* is the most important vector for ZIKV. This study shows that two strains from China exhibit high levels of midgut infection and highly disseminated infection of salivary glands and ovaries. Both strains can transmit ZIKV to infant mice bitten by infectious mosquitoes. Moreover, the results provide the evidence of transovarial transmission of ZIKV in mosquitoes. The study indicates that the two *Ae*. *aegypti* strains are not only effective transmission vectors but also persistent survival hosts for ZIKV during unfavorable inter-epidemic periods. This function as a reservoir of infection has epidemiological implications that further enhance the risk of potential future outbreaks.

## INTRODUCTION

*Aedes (Stegomyia) aegypti*, also known as the yellow fever mosquito, is widely distributed in tropical and subtropical areas around the world, with habitats between the average 10 °C winter isotherms in the northern and southern hemispheres.^1^ On the basis of its ecological behavior, such as inhabiting human dwellings, indoor artificial container breeding, multiple anthropophilic and daytime blood-feedings, and endophilic resting, *Ae. aegypti* has a close relationship with humans. It is a primary or important vector of yellow fever, dengue fever, chikungunya and Zika. Recent emergence, reemergence and expansion of these infectious diseases has become a global threat, and *Ae. aegypti* plays an important role in spreading viral diseases.

Zika virus (ZIKV), a pathogen of the emerging mosquito-borne viral disease of concern, belongs to the genus *Flavivirus*, of the Flaviviridae family, which was first isolated in sentinel rhesus monkeys in Uganda in 1947.^2^ Experimental studies have confirmed that *Ae. aegypti* is a competent and high-efficient vector of ZIKV, but there is evidence that different strains of *Ae. aegypti* show varying competence for the virus.^3, 4, 5, 6, 7, 8^ ZIKV has been isolated from mosquitoes, including species of *Aedes*, that is, *Ae. aegypti*, *Ae. africanus*, *Ae. albopictus*, *Ae. apicoargenteus*, *Ae. dalzieli*, *Ae. furcifer*, *Ae. taylori*, *Ae. furcifer-taylori*, *Ae. luteocephalus*, *Ae. neoafricanus* and *Ae. vitatus*, in Senegal, Ivory Coast, Burkina Faso, Central African Republic, Uganda and Asia.^9^ The first isolation from *Ae. aegypti* occurred in Malaysia in 1967^10^ and later isolations occurred in the Ivory Coast.^11^ Interestingly, ZIKV was isolated in male adults of *Ae. furcifer*, implying possible transovarial transmission in the field.^12^

Transovarial transmission of arboviruses enables virus persistence in nature and is relevant to the epidemical role in outbreaks of vector-borne disease.^13^ Transovarial transmission in *Aedes* has been researched extensively, especially focusing on dengue and chikungunya viruses. Transovarial transmission of dengue virus was documented in the field, where different serotypes of dengue virus in larvae or male adult mosquitoes were detected in different areas of the world.^14, 15, 16^ A series of laboratory studies confirmed that *Ae. aegypti* and *Ae. albopictus* could transovarially transmit dengue virus.^13, 17, 18, 19^ Detection of chikungunya virus in wild mosquito populations has also implied transovarial transmission in the field.^20, 21^ Recent experiments have revealed that this type of transmission can also occur in the laboratory.^22, 23^ The current study reports transmission experiments for *Ae. aegypti* to analyze its competence and to confirm transovarial transmission of ZIKV.

## MATERIALS AND METHODS

### Ethics statement

All experimental protocols involving animals were approved by the Laboratory Animal Center, State Key Laboratory of Pathogen and Biosecurity, Beijing Institute of Microbiology and Epidemiology IACUC (Institutional Animal Care and Use Committee; BIME 2011-09). The study of animals was carried out in strict accordance with the recommendations in the Guide for the Care and Use of Laboratory Animals of the National Institutes of Health.

### Mosquitoes

Two strains of *Ae. aegypti* were used in experimental infection. The HK strain was collected from Haikou City, Hainan province, and reared in the laboratory. The RL strain was collected from Ruili City, Yunnan province, and reared in the laboratory. Mosquitoes were maintained under standard insectary conditions at (26±1) °C and (75±5)% relative humidity (RH), with a photoperiod of 14 h:10 h of light:dark (L:D) cycles. Prior to the infectious feed, adult mosquitoes were provided with 8% sugar water.

### Virus

The contemporary ZIKV strain SZ01 (GenBank No. KU866423) was used to expose the mosquitoes. This virus was originally isolated from the blood of a patient who returned from Samoa to China in 2016.^24^ The stock virus used in the current study was passaged twice in C6/36 cells prior to the infectious feed.

### Oral infection of mosquitoes

Five-day-old female mosquitoes were starved for 12 h prior to the infectious blood meal. The blood meal consisted of 1:1 mouse blood and virus suspension. Oral infections with ZIKV were performed with a virus titer of 3 × 10^5^ plaque-forming unit per mL, verified by titration in a standard plaque assay.^25^ Mosquitoes were fed with an infectious blood meal warmed to 37 °C using a Hemotek membrane feeding system housed in a feeding chamber. After 30 min of blood feeding, mosquitoes were cold-anesthetized, and fully engorged females were transferred to 300 mL plastic cups, maintained at (29±1) °C and (75±5)% RH with a 14 h/10 h L:D cycle, and provided with 8% sugar water.

### Mosquito processing

To determine the ZIKV infection and dissemination rates in *Ae. aegypti*, 10–15 female mosquitoes were sampled at 2, 4, 6, 8, 10, 12, 16, 20 and 24 days post exposure (dpe). To prevent cross-contamination of virus between the midguts, salivary glands and ovaries of each mosquito, these organs were carefully dissected using fresh dissecting needles, and the organs were iteratively rinsed in phosphate-buffered saline three times each. The midguts, salivary glands and ovaries from each mosquito were individually transferred to 1.5 mL microtubes containing 100 μL of Dulbecco's modified Eagle's medium (DMEM; GIBCO, Invitrogen, Beijing, China) supplemented with 2% fetal bovine serum (FBS). These organs were homogenized using 5 mm stainless steel grinding balls (Next Advance, Averill Park, NY, USA) in a Bullet BlenderTM 24 mixer mill (Next Advance) set at frequency of 12/s for 1 min. All dissecting needles were dipped in 80% ethanol, burned and cleaned before reuse.

### Transmission experiments

To determine the ability of *Ae. aegypti* to transmit ZIKV, nine 1-day-old mice were placed in a cage containing ~100 mosquitoes bred with virus blood at 8 dpe. After exposing mice to the mosquitoes for 1 h, the infant mice were removed from the cage, segregated and reared in the lab. The blood-engorged mosquitoes were removed from the cage using CO_2_ anesthesia. The salivary glands of each mosquito was dissected and individually transferred to 1.5 mL microtubes containing 100 mL of DMEM (GIBCO, Invitrogen) supplemented with 2% FBS. Three infant mice were killed at days 4, 7 and 10 post feeding, and their brains were dissected. A weight of 100 mg tissue per brain was transferred to 1.5 mL microtubes containing 100 mL of DMEM (GIBCO, Invitrogen) supplemented with 2% FBS.

### Transovarial transmission experiments

To determine ZIKV transovarial transmission rates (TRs) from mother to offspring in mosquitoes, the eggs of the first gonotrophic cycle and the second gonotrophic cycle were collected.

The eggs from the first gonotrophic cycle were collected at 4–5 dpe, and 29 and 25 pools of eggs (30 eggs per pool) and 35 and 31 salivary glands of emerged females from the Hainan and Yunnan strains of *Ae. aegypti* were collected, respectively. They were transferred to 1.5 mL microtubes containing 100 mL of DMEM (GIBCO, Invitrogen) supplemented with 2% FBS and stored at −80 °C before virus detection. Parts of the parent female mosquitoes used for transovarial transmission were also selected and screened individually following the same method.

Females that laid eggs were fed mouse blood, and eggs from the second gonotrophic cycle were collected on days 4–5 post feeding. Then, 24 and 23 pools of eggs (30 eggs per pool) and 47 and 43 salivary glands of emerged females from the Hainan and Yunnan strains of *Ae. aegypti* were collected, respectively. They were transferred to 1.5 mL microtubes containing 100 mL of DMEM (GIBCO, Invitrogen) supplemented with 2% FBS and stored at −80 °C before virus detection. Parts of parent female mosquitoes used for transovarial transmission were also selected and screened individually following the same method.

### Detection of virus

Total RNA was isolated from midguts, salivary glands and ovaries of mosquitoes, the brains of infant mice, and the eggs pools and salivary glands of the emerged females and parent females using the QIAamp Viral RNA Mini kit (Qiagen, Hilden, Germany) following the manufacturer's recommendations. ZIKV in these organs was detected using the One-Step Detection kit for Zika Virus RNA (DaAn Gene, Guangzhou, China), and PCR was conducted as previously described.^26^

The number of viral RNA copies was calculated by generating a standard curve from RNA isolated from uninfected mosquitoes by titrating with a known amount of seed virus, the titer of which was determined using a plaque assay. The amount of RNA is expressed as log_10_ RNA copies per mL. The standard RNA used in the nucleic amplification assays was extracted from virus dilutions of a known titer as determined by a plaque assay.

### Infection and transmission analysis

The infection rate (IR) of midguts, salivary glands and ovaries on each sampling day was calculated by dividing the number of infected midguts, salivary glands and ovaries by the total number of mosquitoes tested. Transmission was confirmed by the appearance of brain virus-positive infant mouse brains from mice bitten at least once by infected mosquitoes. The TR was calculated by dividing the number of mosquitoes with infected saliva by the number of mosquitoes with a disseminated infection (that is, midgut-/salivary gland-/ovary-positive). The transmission efficiency represents the proportion of mosquitoes with infectious saliva among the total number of mosquitoes tested.

### Transovarial transmission analysis

Transovarial transmission ability was measured by the same methods for the first and second gonotrophic cycles. The IR of egg-laying females or the salivary glands of newly emerged females was calculated by dividing the number of ZIKV-positive individuals by the total number of individuals tested. The IR of eggs was calculated by dividing the number of ZIKV-positive pools by the total number of pools tested. On the basis of results of pool testing, the minimum infection rate (MIR), calculated from the ratio of the number of positive pools to the total number of individuals (eggs) tested, was used to estimate IRs.

**Figure 1:**
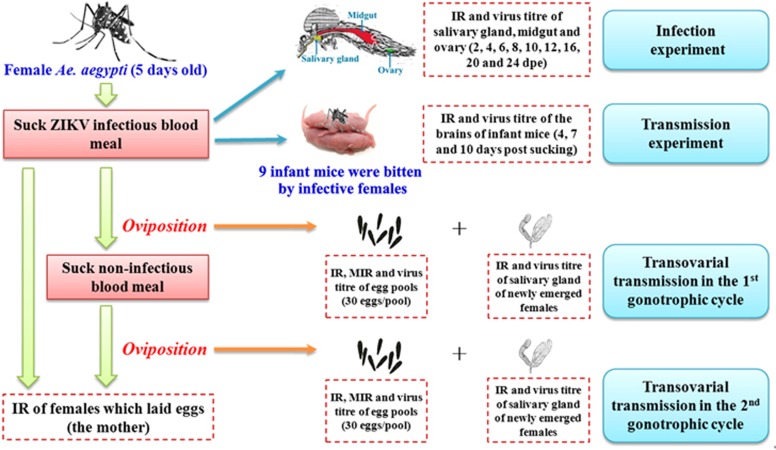
Schematic representation of the experimental design.

A schematic representation of the experimental design and infection, transmission, and transovarial transmission is shown in Figure 1.

### Statistical analysis

All statistical tests were conducted using SPSS 13.0 software (IBM, Armonk, NY, USA). Rates were compared using Fisher's exact test. Kruskal–Wallis tests were used to determine differences in viral titers for the two mosquito strains at different times. *P*-values >0.05 were considered non-significant.

## RESULTS

### Oral susceptibility of two *Ae. aegypti* strains to ZIKV

**Figure 2:**
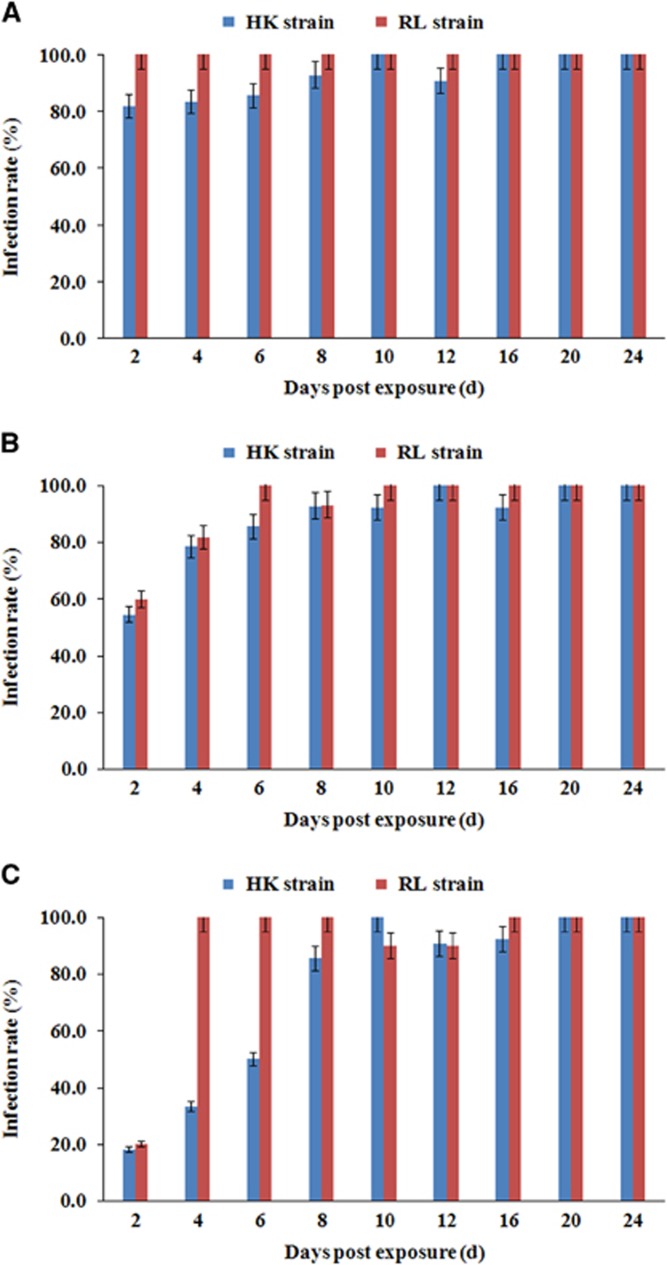
ZIKV infection rates of midguts (**A**), salivary glands (**B**) and ovaries (**C**) in two *Ae.* *aegypti* strains at different dpe to the blood meal. Eleven to fourteen mosquitoes were sampled per day. Error bars represent confidence intervals (95%).

From 2 dpe onwards, after the blood meal had been completely digested, ZIKV could be detected in mosquitoes on each sampling day and increased rapidly from 2 to 8 dpe (Figure 2). The virus must pass through the midgut barrier and spread to the salivary glands and ovaries to cause the infection. Therefore, the time to detect the virus in the salivary glands and ovaries was longer than the time to detect it in the midguts.

When examining IRs, there was no significant difference between the *Ae. aegypti* HK strain and the RL strain (*P*>0.05), except in the IRs of ovaries at 4 and 6 dpe (*P*<0.05). The IR of salivary glands had already reached >50% at 2 dpe. At 8 dpe, the IR of salivary glands for both strains had increased to >90% and remained between 90% and 100% as late as 24 dpe. A high IR value of >80% was maintained in the midguts of the two *Ae. aegypti* strains after 2 dpe. Particularly in the RL strain, the IR in the midguts was 100% from 2 to 24 dpe. There was a slight difference in the ovary IRs: although the difference was not significant at day 2 dpe, the increasing trend of ovarian infection was faster in the RL strain than in the HK strain at 4 and 6 dpe.

### ZIKV titers in the midguts, salivary glands and ovaries of mosquitoes

**Figure 3:**
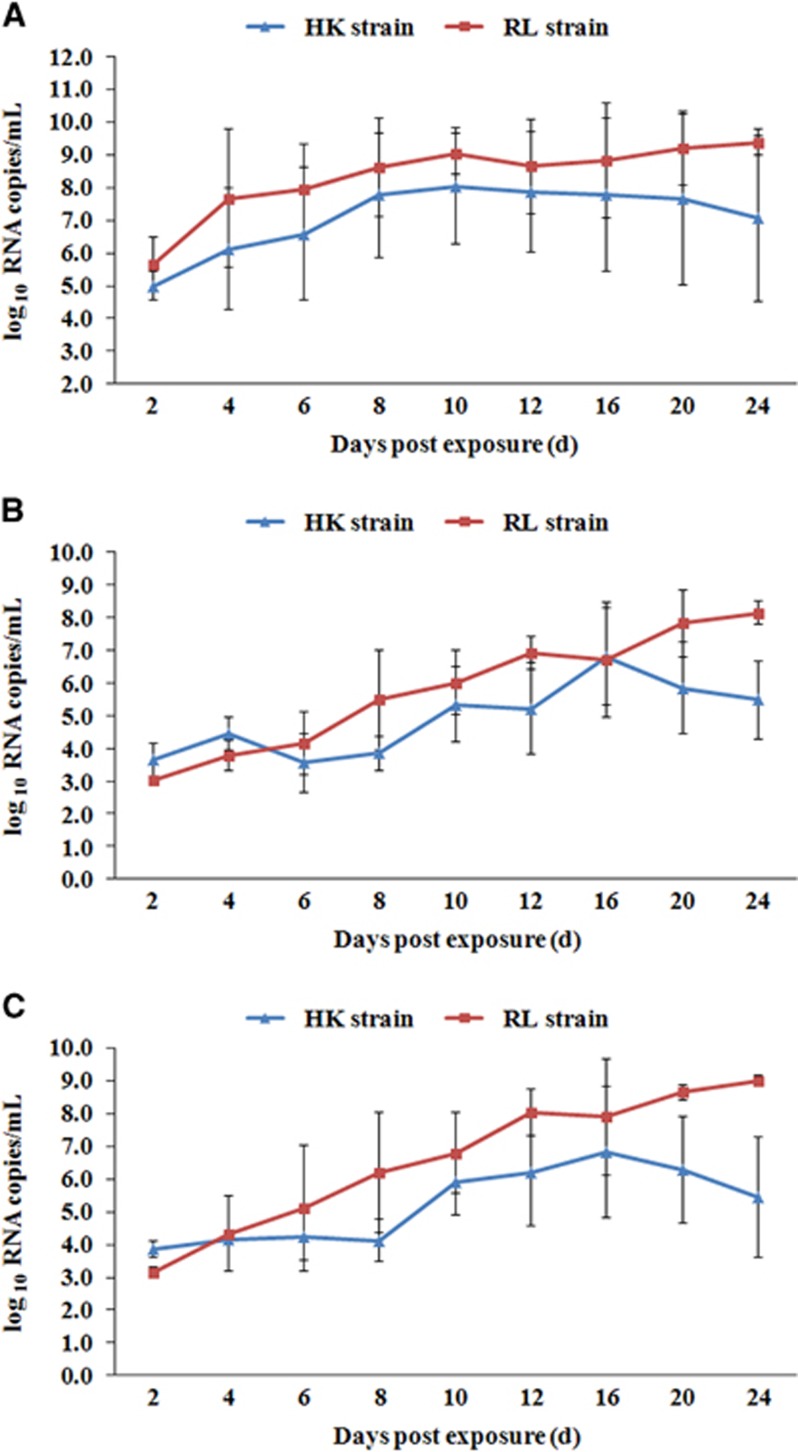
Quantification of Zika viral RNA copies by real-time PCR with reverse transcription detected in midguts (**A**), salivary glands (**B**) and ovaries (**C**) of two *Ae.* *aegypti* strains at different dpe. Error bars represent sd.

Figure 3 presented the estimated average ZIKV titers of the midguts, salivary glands and ovaries of infected females at different dpe. For the RL strain over time, with small fluctuations, the average virus titers of ZIKV in salivary glands, midguts and ovaries exhibited upward trends until 24 dpe. Even at 24 dpe, with average values of 8.14±0.36 log_10_ RNA copies per mL in the salivary glands, 9.38±0.40 log_10_ RNA copies per mL in the midguts and 9.01±0.16 log_10_ RNA copies per mL in ovaries, the ZIKV titer was still increasing. For the HK strain, the highest titers were detected at day 16 dpe in salivary glands and ovaries, with average values of 6.79±1.47 log_10_ RNA copies per mL and 6.82±2.01 log_10_ RNA copies per mL, respectively, and at day 10 dpe in the midguts, with an average value of 8.05±1.78 log_10_ RNA copies per mL.

Thus, the viral titer in the RL strain was higher than that in the HK strain. The overall trend, shown in Figure 2, is that except at 2 dpe, the viral titer of salivary glands in the RL strain was significantly higher than that in the HK strain, especially at 8, 12, 20 and 24 dpe (*P*<0.05). This trend was also true in the ovaries at 8, 12, 20 and 24 dpe. In the midguts, although the viral titer in the RL strain was consistently higher than that in the HK strain on the curve, this difference was significant only at 20 and 24 dpe Combined with Figures 2A–2C, ZIKV proliferation presents distinct trends in different *Ae. aegypti* strains. The virus continued to proliferate in the RL strain at 24 dpe. In the HK strain, the virus titers began to decline at 16 dpe, indicating lower competence than the RL strain.

### Transmission of ZIKV by two *Ae. aegypti* strains

Infant mice bitten by infectious mosquitoes can be infected with the virus, which can break through the blood–brain barrier to replicate in the mouse brain, providing direct evidence that this mosquito species can transmit ZIKV and is a potential vector. More than one red blotch was present on the skin of all nine infant mice after removal from the virus-infected mosquito cage, which indicated that all infant mice were bitten by at least one mosquito. Among 21 and 24 blood-engorged mosquitoes, 19 (IR=90.5%) and 23 (IR=95.8%) had viral RNA in their salivary glands corresponding to the HK strain and RL strain, respectively. At 4, 7 and 10 days after being bitten by infected mosquitoes, the virus titer in infant mouse brains continued to increase, with a significant difference (*P*<0.05) in virus titers at the three sampling times. There was no significant difference between the two *Ae. aegypti* strains at four and seven days post feeding. However, at 10 days post feeding, the average titer in the brains of mice bitten by RL strain females (9.59±0.71 log_10_ RNA copies per mL per mouse brain) was significantly higher than that for mice bitten by HK strain females (8.13±0.57 log_10_ RNA copies per mL per mouse brain; Table 1).

### Transovarial transmission in *Ae. aegypti*

Mosquitoes can transmit ZIKV from mother to offspring, which is direct evidence that this mosquito species can vertically transmit ZIKV. In this study, two *Ae. aegypti* strains demonstrated ZIKV transovarial transmission (Tables 2 and 3). In the first and second gonotrophic cycles, the IR values of oviposited females that were randomly removed from the cages were 100% for the two *Ae. aegypti* strains. There was no significant difference between the IR values of eggs laid in the first and second gonotrophic cycles for the same mosquito strain (*P*>0.05). However, the IR of eggs laid by the RL strain was nearly twice that of eggs laid by the HK strain in the first gonotrophic cycle. The IR of the salivary glands of newly emerged adult mosquitoes was 17.14% in the HK strain and 16.13% in the RL strain in the first gonotrophic cycle. It demonstrated that ZIKV was transovarially transmitted from parents to offspring in *Ae. Aegypti*. For the newly emerged adult mosquitoes, virus infection was significantly different (*P*<0.05) between the two gonotrophic cycles. The IR of the salivary glands of newly emerged females was decreased to 2.13% in the HK strain and 2.33% in the RL strain in the second gonotrophic cycle. There was no significant difference in the IR values of the salivary glands of newly emerged females between the two *Ae. aegypti* strains.

## DISCUSSION

As ZIKV was first isolated in Africa, early infection and transmission experiments have confirmed that *Ae. aegypti* from Nigeria was competent to ZIKV.^3^ Recently, several studies conducted with different geographical strains of *Ae. aegypti* and different ZIKV isolates have indicated that *Ae. aegypti* in Singapore is susceptible to ZIKV, with high midgut IRs and salivary gland dissemination rates observed by day 5 after the infectious blood meal.^5^ Two populations of *Ae. aegypti* from Senegal showed high midguts IRs but low dissemination rates, and ZIKV RNA was not detected in the saliva of these *Ae. aegypti*.^6^ High IRs, but lower disseminated infection and TRs, were observed in five *Ae. aegypti* populations from America.^7^ This study showed that two strains from the Hainan and Yunnan provinces in China exhibited high infection of midguts (81.82%–100.00% in the HK strain and 100.00% in the RL strain) and high disseminated infection of salivary glands and ovaries. Transmission confirmed that both *Ae. aegypti* strains can transmit ZIKV to infant mice bitten by infectious mosquitoes, implying that *Ae. aegypti* from China is competent to ZIKV. Experimental infection, transmission and transovarial transmission results showed that susceptibility to ZIKV and transovarial transmission ability were higher in the RL strain than in the HK strain. These differences in susceptibility may be due to geographical variations in the strains.

Transovarial transmission is considered a primary means by which some arboviruses are maintained despite adverse environmental conditions.^27^ Because *Aedes* eggs are desiccation-resistant, they can survive for long periods, leading to the persistence of arbovirus in the eggs.^28^ In previous research, dengue virus, chikungunya virus, West Nile virus, Ross River virus, Sindbis virus and Western Equine Encephalitis virus have been isolated from adult *Aedes* species reared from larvae collected from natural habitats, confirming the existence of natural transovarial transmission.^29^ Thangamani's recent research demonstrated that ZIKV could be vertically transmitted from infected adults to the F_1_ offspring through intrathoracic inoculation in *Ae. aegypti*.^30^ The results of this study show the evidence of transovarial transmission of ZIKV in mosquitoes by a natural oral route of infection. Moreover, this report is the first evidence of mosquito-borne ZIKV in the first gonotrophic cycle after an infectious blood meal, although several other mosquito-borne viruses have been found in the second gonotrophic cycle post infectious blood meal, including dengue virus in *Ae. aegypti*,^17, 19^ chikungunya virus in *Ae. aegypti*,^22^ West Nile virus in *Cx. pipiens pipiens*,^31^ La Crosse virus in *Ae. triseriatus*^32^ and yellow fever virus in *Ae. aegypti*.^33^

ZIKV was detected in the ovaries of infectious female mosquitoes on the second day post infectious blood meal, with 100% IR in ovaries at 4 dpe in the RL strain and 10 dpe in the HK strain. This result implies that eggs were infected with ZIKV in the first gonotrophic cycle. This result is different from that for chikungunya virus, for which viral dissemination started at day 3 post infection and peaked on day 10. The MIR values achieved for eggs were 1.15% and 2.13% in HK and RL strain, respectively. Such high MIR values in transovarial transmission experiments were also described previously for Japanese encephalitis, West Nile and chikungunya viruses.^22, 27, 34, 35, 36^ These high MIR values are likely to be an indicator of potential outbreaks.

The introduction of ZIKV to the Americas and its rapid spread across the continent is likely attributable to the globalization of trade and travel, as well as the ability of local *Aedes* to disseminate and transmit ZIKV. As the first case of laboratory-confirmed ZIKV infection in China on 6 February 2016,^37^ more cases have been continuously reported. In China, *Ae. aegypti* is mainly distributed in the Leizhou Peninsula of Guangdong province, Hainan province^38^ and the Yunnan border area near Myanmar and Lao.^39^ On the basis of the high competence for transmission of *Ae. aegypti* to ZIKV demonstrated in this study, Guangdong, Hainan and Yunnan province are high-risk areas for potential ZIKV transmission. Moreover, the ability of transovarial transmission of *Ae. aegypti* indicates that this species can be a persistent survival host of ZIKV during unfavorable inter-epidemic periods and also a transportable reservoir of infection. This finding has epidemiological implications that demonstrate enhanced risk of potential future outbreaks.

## Figures and Tables

**Table 1:** Positive percentages and viral titers in the brains of infant mice bitten by infectious mosquitoes

**Strain**	**Number of mosquitoes**^a^	**Number of mice tested**	**Number of positive mice**	**Positive percent (%)**	**Average virus titer of mouse brain (log**_**10**_ **RNA copies per mL)**^b^		
					**4d**	**7d**	10d*
HK	21	9	9	100.00	5.86±0.43	7.84±1.18	8.13±0.57
RL	24	9	9	100.00	6.92±1.07	8.50±0.24	9.59±0.71

Abbreviations: days, d; Zika virus, ZIKV.

a Number of blood-engorged mosquitoes removed from the cage by CO_2_ anesthesia after blood-sucking. All salivary glands of these mosquitoes were ZIKV-positive.

b At 10 days post feeding, the average virus titer in the brains of infant mice bitten by RL strain females was significantly higher than that for those bitten by HK strain females.

* At 10 days post-feeding, the average titre in the brains of mice bitten by RL strain females was significantly higher than that for mice bitten by HK strain females.

**Table 2:** IR, MIR and viral titers of oviposited females, eggs and newly emerged females in the first gonotrophic cycle of infectious *Ae.* *aegypti*

**Exp.**	**Strain**	**Number tested**	**Number positive**	**IR**^a^ **(%)**	**MIR**^b^ **(%)**	**log**_**10**_ **RNA copies per mL**
Oviposited females^c^	HK	14	14	100	—	7.48±1.31
	RL	13	13	100	—	7.70±0.61
Eggs (30 per pool)	HK	29	10	34.48	1.15	3.16±0.25
	RL	25	16	64	2.13	3.85±0.67
Salivary gland of newly emerged females^d^	HK	35	6	17.14	—	5.04±0.91
	RL	31	5	16.13	—	4.25±0.35

Abbreviation: Zika virus, ZIKV.

a IR (infection rate) is calculated as the ratio of the number of ZIKV-positive individuals or pools to the total number of individuals or pools tested.

b MIR (minimum infection rate) is the ratio of the number of positive pools to the total number of individuals (eggs) tested.

c Oviposited females were mosquitoes that laid eggs in the first gonotrophic cycle. Ten to fifteen individuals were randomly removed from cages and analyzed.

d Eggs obtained from the first gonotrophic cycle were reared under standard laboratory conditions through the larvae and pupae stages until a new generation of adults emerged. The salivary glands of newly emerged females were dissected and analyzed.

**Table 3:** IR, MIR and viral titers of oviposited females, eggs and newly emerged females in the second gonotrophic cycle of infectious *Ae.* *aegypti*

**Exp.**	**Strain**	**Number tested**	**Number positive**	**IR**^a^ **(%)**	**MIR**^b^ **(%)**	**log**_**10**_ **RNA copies per mL**
Oviposited females^c^	HK	14	14	100	—	9.48±1.12
	RL	10	10	100	—	8.05±2.77
Eggs (30 per pool)	HK	24	13	54.17	1.81	4.41±0.82
	RL	23	12	52.17	1.74	5.19±0.71
Salivary gland of newly emerged females^d^	HK	47	1	2.13	—	4.77±0.00
	RL	43	1	2.33	—	3.45±0.00

Abbreviation: Zika virus, ZIKV.

a IR (infection rate) is calculated as the ratio of the number of ZIKV-positive individuals or pools to the total number of individuals or pools tested.

b MIR (minimum infection rate) is the ratio of the number of positive pools to the total number of individuals (eggs) tested.

c Oviposited females were mosquitoes that laid eggs in the second gonotrophic cycle. Ten to fifteen individuals were randomly removed from cages and analyzed.

d Eggs obtained from the second gonotrophic cycle were reared under standard laboratory conditions through the larvae and pupae stages until a new generation of adults emerged. The salivary glands of newly emerged females were dissected and analyzed.
